# Evaluation of ASCA IgA/IgG seropositivity, fecal calprotectin levels and NOD2 gene polymorphisms in patients with hidradenitis suppurativa: A prospective case-control study

**DOI:** 10.1016/j.jdin.2025.11.029

**Published:** 2025-12-19

**Authors:** Mahmut Esat Tanrıbilir, Ahmet Burhan Aksakal, Özlem Gülbahar, Gizem Yaz Aydın, Gülsüm Kayhan, Tarkan Karakan, Esra Adışen

**Affiliations:** a Department of Dermatology, Ministry of Health Dr. Vefa Tanır Ilgın State Hospital, Konya, Turkey; b Department of Dermatology, Gazi University Faculty of Medicine, Ankara, Turkey; c Department of Biochemistry, Gazi University Faculty of Medicine, Ankara, Turkey; d Department of Biochemistry, Pursaklar State Hospital, Ankara, Turkey; e Department of Medical Genetics, Gazi University Faculty of Medicine, Ankara, Turkey; f Division of Gastroenterology, Department of Internal Medicine, Gazi University Faculty of Medicine, Ankara, Turkey

**Keywords:** ASCA seropositivity, biomarkers, fecal calprotectin, Hidradenitis suppurativa, intestinal inflammation, NOD2 gene polymorphisms

*To the Editor:* Hidradenitis suppurativa (HS) is a chronic inflammatory disorder characterized by painful nodules, abscesses, sinus tracts, and scarring with incompletely understood pathogenesis.^1^ Progression to advanced disease and the role of B cells in HS etiopathogenesis remain under active investigation. Tertiary lymphoid organs, containing B cells, have been observed in chronic HS lesions, implicating these cells in fibrotic processes.^2^ Additionally, growing evidence suggests a link between HS and intestinal inflammation, through a shared gut-skin immune axis. Biomarkers reflecting mucosal immune activation, such as anti-*Saccharomyces cerevisiae* antibodies (ASCAs), fecal calprotectin, and several *NOD2* gene variants, may help elucidate this connection^3^, ^4^, ^5^ (Supplementary Table I, available via Mendeley at https://data.mendeley.com/datasets/dkny7zz5xc/1). We prospectively investigated these biomarkers in patients with HS and healthy controls to explore their potential role in HS pathogenesis, severity, and chronicity.

This prospective, single-center case-control study was conducted at Gazi University Dermatology Unit (Ankara, Türkiye) between January 2022 and January 2023, following approval from the institutional ethics committee (No. 46, January 24, 2022). Written informed consent was obtained from all participants. Seventy patients with HS and 50 age- and sex-matched healthy volunteers were enrolled, all were of Turkish origin. Participants with symptoms of inflammatory bowel disease (IBD) or systemic inflammatory disorders were excluded, and family history of HS was recorded. Serum ASCA IgA/IgG levels, fecal calprotectin, and 4 *NOD2* gene polymorphisms (R702W, G908R, IVS8+158, P268S) were analyzed. Laboratory procedures are detailed in Supplementary Text 1, available via Mendeley at https://data.mendeley.com/datasets/dkny7zz5xc/1. Disease severity was graded using the Hurley stage and HS-PGA scales. Statistical analyses were performed using SPSS v22, and a *P*-value <.05 was considered significant. The study workflow is summarized in Supplementary Fig 1, available via Mendeley at https://data.mendeley.com/datasets/dkny7zz5xc/1. Patients with HS were categorized with Canoui-Poitrine, Van der Zee/Jemec, and Martorell classification systems phenotypically.

We found that ASCA IgA seropositivity was significantly higher in patients with HS compared with the control group (Table I). Patients with Hurley stage III had significantly higher ASCA IgA seropositivity compared to patients with Hurley stage I and II. Additionally, patients with ASCA IgA seropositivity had longer disease duration and a greater number of affected anatomical sites, suggesting a possible link between mucosal immune activation and HS chronicity (Table II). In contrast, we did not find a significant association between ASCA IgG seropositivity and Hurley stages. In addition, no statistically significant association was found between different phenotypical subgroups and ASCA IgA or IgG seropositivity.

**Table I:** Comparison of ASCA antibody status, *NOD2* gene polymorphisms, fecal calprotectin levels between patients with HS and control group

Characteristics	Patients with HS group (*n* = 70)	Control group (*n* = 50)	*P* value
Gender			
Female (%)	38.6%	40%	
Male (%)	61.4%	60%	.87
Age (y) median IQR	37 (26-48)	36.5 (28.75-46.25)	.62
BMI (kg/m^2^), median (IQR)	28.91 (24.81-32.11)	24.89 (22.26-28)	<.01
ASCA IgA seropositivity (%)	20.6%	4%	.01
ASCA IgG seropositivity (%)	26.5%	12%	.054
FC > 150 μg/g (%)	23.5%	0	<.01
*NOD2* gene loci			
IVS8+158 (rs5743289)			
C/C	74.3%	78%	
C/T	24.3%	22%	
T/T	1.4%	0	.90
P268S (rs2066842)			
C/C	64.3%	64%	
C/T	35.7%	36%	.97
R702W (rs2066844)			
C/C	100%	100%	1.00
G908R (rs2066845)			
G/G	98.6%	96%	
C/G	1.4%	4%	.57

**Table II:** Comparison of clinical features between ASCA IgA seropositive and seronegative HS patients

Characteristic	ASCA IgA seronegative (*n* = 54)	ASCA IgA seropositive (*n* = 14∗)	*P* value
Gender			
Female (*n*) %	22 (40.7%)	4 (28.6%)	
Male (*n*) %	32 (59.3%)	10 (71.4%)	.40
Age median (IQR)	36.5 (25.5-48)	36 (25.75-46.25)	.78
BMI kg/m^2^ median (IQR)	28.70 (24.1-31.4)	30.4 (27.01-36.46)	.19
Duration since diagnosis (y) median (IQR)	2 (1-5)	5 (2-7.25)	.02
Active smoking (*n*) %	30 (55.6%)	8 (57.1%)	.99
Involved anatomic sites (*n*) median IQR	3 (2-4)	4 (3-5)	.02
Fermented food as a triggering factor (*n*) (%)	7 (13%)	3 (21.4%)	.42
Hurley stage *n* (%)			
Stage I	15 (27.8%)	2 (14.3%)	
Stage II	25 (46.3%)	3 (21.4%)	
Stage III	14 (25.9%)	9 (64.3%)	.02
Disease phenotypes			
Canoui-Poitrine classification (*n*) (%)			
Axillary/mammary	26 (48.2%)	6 (42.9%)	
Follicular	8 (14.8%)	3 (21.4%)	
Gluteal	20 (37.0%)	5 (35.7%)	.85
Martorell classification (*n*) (%)			
Follicular	5 (9.3%)	0 (0%)	
Inflammatory	26 (48.1%)	9 (64.3%)	
Mixed	23 (42.6%)	5 (35.7%)	.53
FC >150 μg/g (%)	17.1%	44.4%	.09

∗ ASCA IgA results were unavailable for 2 patients due to inadequate serum samples, analyses were conducted on 68 patients.

Elevated fecal calprotectin (FC) levels were observed in 23% of patients with HS, while none of the controls exceeded this threshold, indicating a potential subclinical inflammation in a subset of patients with HS (Supplementary Table III, available via Mendeley at https://data.mendeley.com/datasets/dkny7zz5xc/1). One patient with markedly increased FC levels (>1000 μg/g) was later diagnosed with Crohn disease.

Analysis of 4 *NOD2* gene polymorphisms (R702W, G908R, IVS8+158, P268S) revealed no significant differences between patients with HS and control group, nor associations with clinical features, ASCA seropositivity, or elevated FC levels (Supplementary Table IV, available via Mendeley at https://data.mendeley.com/datasets/dkny7zz5xc/1).

Although limited by sample size, our findings suggest that ASCA IgA antibodies may serve as potential biomarkers of HS chronicity and disease burden, highlighting the need for larger studies investigating gut-related immune dysregulation in HS.

## Conflicts of interest

All authors have read and approved the manuscript, and there are no conflicts of interest to declare. The manuscript has not been published elsewhere and is not under consideration by any other journal.
